# Conceptual trait associations predict impressions of highly variable faces

**DOI:** 10.1111/bjop.70031

**Published:** 2025-09-24

**Authors:** Barbora Illithova, Andrew W. Young, Mingyuan Chu, Clare A. M. Sutherland

**Affiliations:** ^1^ School of Psychology, King's College University of Aberdeen Aberdeen UK; ^2^ Department of Psychology University of York York UK; ^3^ School of Psychological Science University of Western Australia Crawley Western Australia Australia

**Keywords:** conceptual trait associations, cross‐cultural, dynamic interactive theory, face impressions, individual differences, person perception

## Abstract

People form consequential trait judgements from seeing others' faces. The influential dynamic interactive theory suggests that trait judgements reflect the combined use of visual cues from faces (e.g. smiling looks trustworthy) with individuals' own conceptual trait associations (e.g. believing trustworthy people are also kind), thus far supported for impressions of highly constricted neutral faces in the US cultural context. Here, we provide a stringent new test of the dynamic interactive theory by examining whether conceptual trait associations predict impressions of highly variable everyday faces, within and across cultures and individuals. Study 1 shows that conceptual trait associations predict impressions of highly variable everyday faces in British perceivers. Study 2 demonstrates that British and Chinese perceivers' conceptual trait associations (expressed in English and Mandarin, respectively) predict impressions of highly variable White and Asian faces similarly. Study 3 finds that individuals' conceptual trait associations predict their impressions of highly variable face images. Together, we show for the first time that conceptual trait associations predict impressions even when faces provide rich visual cues and extend this understanding beyond Western perceivers, faces and languages. Our findings thus offer independent support for dynamic interactive theory in naturalistic impressions across cultures.

## BACKGROUND

Despite their limited accuracy (Foo et al., 2022; Sutherland & Young, 2022), a wide range of impressions can be inferred from an image of an unfamiliar person's face within a single glance (Willis & Todorov, 2006). These face impressions can influence important social decisions, such as electoral success (Olivola et al., 2012), career success (Baert, 2017; Rule & Ambady, 2008) or even the severity of criminal sentencing (Wilson & Rule, 2015). Visual cues have long been shown to elicit specific face impressions commonly across perceivers (e.g. smiling faces tend to elicit perceived trustworthiness; Oosterhof & Todorov, 2008; Vernon et al., 2014). Yet, more recent research suggests that perceivers' individual experiences and environments also play an important role in the impressions they form (e.g. FeldmanHall et al., 2018; Germine et al., 2015; Sutherland, Burton, et al., 2020; see Sutherland & Young, 2022 for a review). Critically, the same face image can lead to impressions that differ from one perceiver to another, especially when the available facial cues are highly varied (e.g. Germine et al., 2015; Hehman et al., 2017). These idiosyncratic judgements suggest that it is crucial to understand factors beyond visual cues to fully understand face impressions.

An influential model that explicitly considers the role of the perceiver in impressions is the dynamic interactive theory (Freeman et al., 2020; see also Over & Cook, 2018; Wang et al., 2016; for related person perception models). Dynamic interactive theory argues that face impressions emerge from an integration of top‐down factors (e.g. task demands), facial cues (e.g. facial morphology), and, crucially, perceivers' unique conceptual associations. For example, if someone has a positive association between attractiveness and trustworthiness, when encountering someone who appears attractive, they may perceive the face to *look* more trustworthy (and/or vice versa). A recent but growing body of evidence has now shown that these kinds of conceptual associations predict impressions from faces in groups of perceivers (e.g. Bin Meshar et al., 2022; Hong & Freeman, 2024; Stolier et al., 2018, 2020), individuals (Stolier et al., 2018, 2020) and can even causally influence face judgements (Stolier et al., 2020), thus giving them a likely role in individual differences in impressions.

**FIGURE 1:**
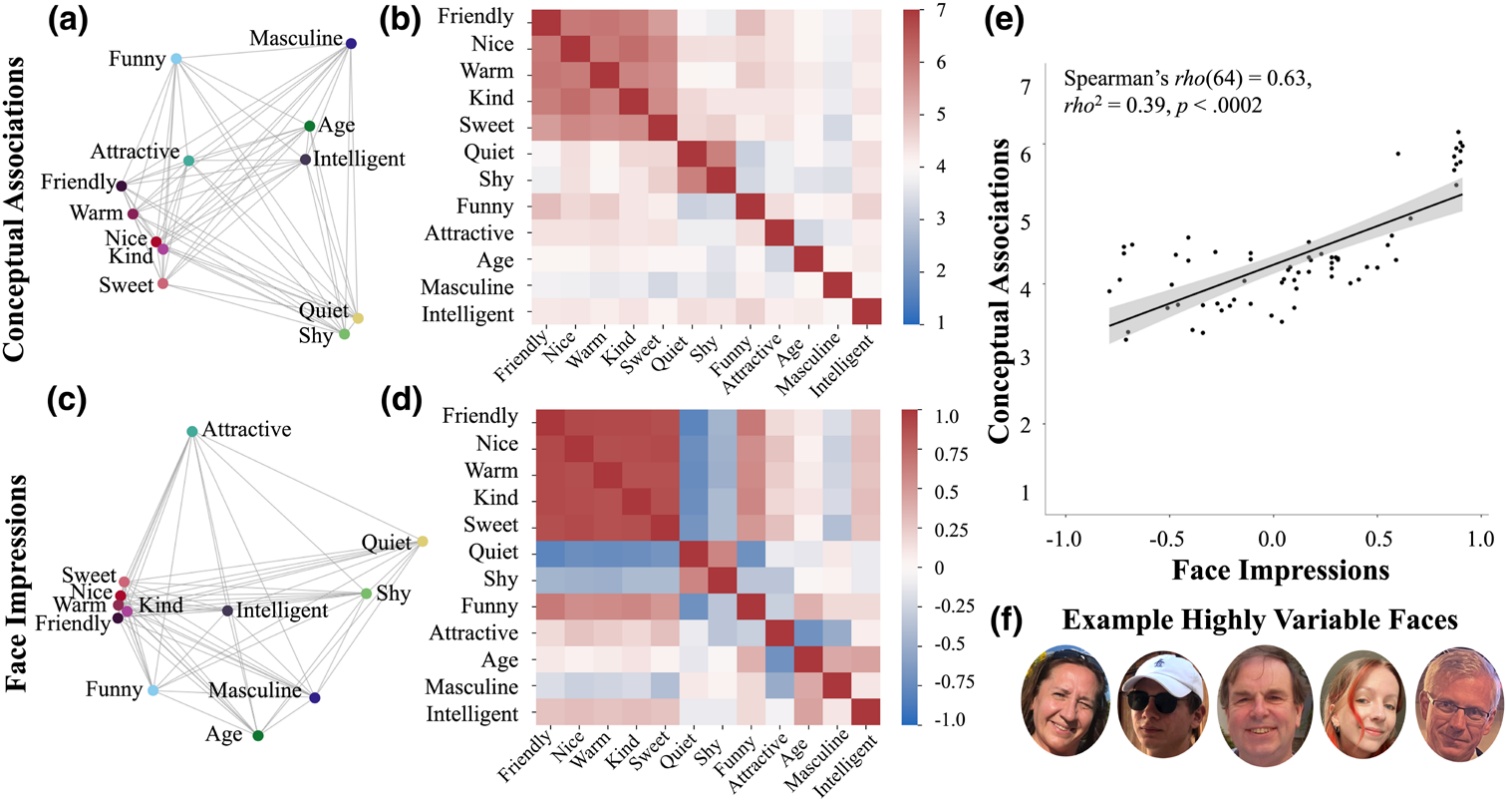
British conceptual associations positively predict white face impressions. Multidimensional scaling represents relationships between traits as rated in conceptual associations (a) and face impressions (c). Each coloured coordinate point represents one (the same) trait. Traits that lie closer to each other are also perceived as more similar. Each point in the two‐dimensional MDS plot reflects the position of the trait on the first two dimensions extracted from MDS (approachability and dimension two for conceptual associations, approachability and youthful‐attractiveness for face impressions). (b) Conceptual associations matrix (values represent the average similarity between traits, 7 = very similar, 1 = not at all similar), traits ordered based on their importance to face impressions from prior study of Sutherland et al. (2018). Full matrix is used for visualization and only the values in the lower triangle without the diagonal (unique trait similarities) were used for the analyses. (d) Face impression matrix from Sutherland et al. (2018; values depict Pearson's correlation coefficient). As above, only unique values (lower triangle without the diagonal) were used for the analyses. (e) Scatterplot illustrates the relationship between conceptual associations and face impressions based on the lower triangle of matrices in b and d used in RSA (raw values, no diagonal). Line of best fit and 95% confidence intervals plotted. (f) Example of White highly variable face images. Unlike standardized images, these images vary highly on, for example, age, expression, hair, makeup, lighting, or viewpoint.

However, while these studies have been seminal in furthering our understanding of impressions from faces, they primarily utilized highly standardized images of emotionally neutral and young White faces, judged by US perceivers in the English language. This approach leaves two crucial theoretical questions unanswered. First, it is unclear if the link between trait cognitions and perceptions of faces remains with more variable images, as the images used in these seminal studies were heavily constrained. They therefore lacked variability in many of the rich visual cues present in everyday face images that convey important aspects of impression formation, such as age, emotion, makeup, or viewpoint (e.g. Collova et al., 2019; Hester & Hehman, 2023; Sutherland et al., 2017; Twele & Mondloch, 2022; see Figure 1f for examples of highly variable images). Note that increasing such naturalistic variation is not trivial but instead has led to changes in the theoretical understanding of key dimensions of impressions (see Sutherland & Young, 2022 for a review). Whilst spontaneous impressions of highly constricted standardized young White face images yield impressions of valence/trustworthiness and dominance (Oosterhof & Todorov, 2008), spontaneous impressions reflect niceness and shyness in children's faces (Collova et al., 2019), sternness and confidence in older adults' faces (Twele & Mondloch, 2022), and, in addition to valence/trustworthiness and dominance, youthful‐attractiveness emerges for highly variable everyday faces (Sutherland et al., 2013, 2018). Crucially, we do not know how such highly variable everyday face images influence the relationship between conceptual associations and face impressions. On the one hand, conceptual associations might be expected to be *less* influential when the visual cues provide richer information for an impression to occur, as the impression formation process may rely more on the abundant visual information provided by the increased face variability (Freeman et al., 2020). On the other hand, however, increased variability may correspondingly increase available stereotypes (Bargh et al., 1996; Chen & Bargh, 1997) and resulting individual differences, suggesting that conceptual associations might be *more* (or at least equally) influential in impressions of such highly variable faces. Indeed, more highly variable faces give rise to greater individual differences in impressions (Hehman et al., 2017), although links with trait concepts have not yet been studied. Thus, it is critical that the dynamic interactive theory and the general importance of conceptual associations are established with more highly variable face images.

Second, it is unclear whether the role of conceptual associations extends beyond Western contexts. According to the dynamic interactive theory, any cultural differences in conceptual associations should also be reflected in people's face impressions (Freeman et al., 2020). That is, although the specific content of conceptual associations or face impressions may differ across cultures (e.g. face judgements of social competence can be positively related to trustworthiness in the United States but not in China: Chen et al., 2016), conceptual associations should still predict face impressions to a comparable extent (i.e. in both cultures, the associations between social competence and trustworthiness should predict face impressions). However, existing studies almost exclusively test White faces and US perceivers using the English language (e.g. Bin Meshar et al., 2022; Hong & Freeman, 2024; Stolier et al., 2018, 2020). Suggestively, one pioneering study of primarily US White perceivers showed that beliefs about specific social groups (e.g. how likely is an attractive Black woman to be trustworthy) predict impressions of highly constricted standardized faces of corresponding social groups (Xie et al., 2021). One other landmark study examined how real personality differences across world regions related to US perceivers' personality trait associations and US perceivers' impressions of White faces (Oh et al., 2022). The more a world region's real personality mapped US perceivers' personality trait associations, the more the world region's real personality predicted US face impressions. Thus, real personality (and, presumably, personality trait associations) within a world region might play a role in face impressions. While suggestive, both studies leave open the direct question of whether conceptual associations predict impressions of faces outside of the United States. If conceptual associations predict perceiver variation in face impressions in a general way, then they should still predict face impressions given more diverse faces, languages, and non‐Western perceivers.

### The current study

Here, in three studies, we tested the relationship between face impressions and conceptual associations at three levels of variability (groups, cultures and individuals) using highly variable face images.

Study 1 addresses whether conceptual associations predict impressions of highly variable everyday faces which provide rich visual cues, such as differences in age, accessories, makeup, lighting, viewpoint or emotional expression, in groups of perceivers on average. Our study provides the first step in establishing the significance of conceptual associations in everyday impression formation. First, we utilize multidimensional scaling (MDS; see also Methods), a dimension‐reduction approach similar to principal components analysis, to compare which underlying dimensions face impressions and conceptual associations share. Second, we employ representational similarity analysis (RSA), used to correlate representations across different modalities without reducing them into dimensions, to quantify the extent to which conceptual associations (i.e. representing beliefs about how traits co‐occur in others) predict correlations between trait impressions as judged in faces (i.e. representing how trait judgements actually co‐occur in faces).

Dimension‐reduction and multivariate approaches are surprisingly rarely used together, despite being highly complementary. Dimension‐reduction approaches informed some of the most influential models of impressions, which seek to understand what fundamental judgements arise when we form impressions (e.g. Lin et al., 2021; Oosterhof & Todorov, 2008; Sutherland et al., 2013). RSA approaches mainly inform a newer wave of person perception research, which aims to describe the mechanisms and dynamics in which multivariate social judgements arise (e.g. Freeman et al., 2020). In favour of RSA, one may argue that the investigation of social judgements should not be restricted to a few dimensions, as these dimensions may depend on the study constraints rather than dimensions' inherent centrality. In favour of dimensional approaches, one may argue that RSA is not concrete enough to capture what is important in the content of impressions. However, these two approaches need not be on opposite ends: by combining RSA with a more traditional dimension‐reduction MDS, we not only investigate how similar conceptual associations and face impressions are, but also which underlying dimensions may drive this similarity. We thus bridge the gap between how these methods have been utilized in impression research thus far for the first time by contextualizing the similarity between concepts and percepts.

In Study 2, we set out to understand whether the relationship between conceptual associations and face impressions exists outside of Western perceivers and White faces. First, following Study 1, we hypothesized that the dimensions of Chinese perceivers' conceptual associations would reflect the dimensions of highly variable Asian face impressions (now in Mandarin; approachability, youth, attractiveness, and capability; Sutherland et al., 2018). Second, we hypothesized that Chinese perceivers' conceptual associations would predict impressions of highly variable Asian faces, as measured via RSA. Finally, we examined whether the extent to which conceptual associations relate to face impressions differs across British and Chinese cultures.

Finally, in Study 3, we assess the relationship between conceptual associations and face impressions across individual perceivers rather than groups. Central to the importance of trait associations is that they have been used to help explain individual perceivers' face impressions (Stolier et al., 2018). If conceptual associations play a role in shaping face impressions, then the more that a given person deems any two traits to be conceptually associated, the more similar their facial judgements of these traits should be (cf. Stolier et al., 2018, 2020). Thus, to provide a stricter final test for the dynamic interactive theory, in Study 3, we recruited a new set of participants who now provided both face impressions and conceptual associations. In this study, participants served as the unit of analysis, with one score each for their conceptual associations and face impressions. We hypothesized that participants' idiosyncratic conceptual associations would correlate with their face impressions, as measured via Spearman's rank correlation. We also aimed to cross‐validate our previous findings at the group level.

Overall, the Dynamic Interactive Theory potentially offers a more comprehensive framework for understanding impression formation, yet it has not been fully tested with highly variable, naturalistic, and diverse face impressions, critical omissions for the breadth of the theory. Together, our three studies provide a novel and comprehensive test of the dynamic interactive theory in more everyday contexts by establishing whether the relationship between conceptual associations and face impressions continues given richer everyday variation in faces, cultures and perceivers.

## STUDY 1

Here, we tested whether conceptual associations predict impressions of faces which provide richer visual cues. We first reasoned that if conceptual associations predict face impressions, then similar underlying dimensions should be reflected in both: specifically, we hypothesized that conceptual associations would cluster together along dimensions found to be important for impressions of such variable faces (approachability, youthful‐attractiveness, and capability; Sutherland et al., 2013, 2018), as measured via multidimensional scaling. Crucially, we hypothesized that there would be a positive relationship between conceptual associations and impressions of highly variable face images, as measured via representational similarity analysis. The study was pre‐registered (https://doi.org/10.17605/OSF.IO/X453B).

### Method

#### Conceptual associations

##### Participants

We recruited 150 participants (based on the original study of conceptual associations, Stolier et al., 2018). Three participants were excluded as they failed to report their English proficiency. Our final sample thus included 147 participants (female = 50%, male = 49%, non‐specified = 1%), with a mean age of 31 years old (SD ± 12.19). Participants were recruited from Testable Minds and compensated $2 for their time. Most of our participants identified as White (78%), followed by Asian (10%), Black (7%), mixed or other ethnicity (8%), or non‐specified (1%). All participants were either native or fluent English speakers and UK residents. Most participants were UK nationals (78%), followed by other European nationality (9%), other world nationality (8%), or non‐specified (5%). The study was approved by the Psychology Ethics Committee of the School of Psychology, University of Aberdeen (PEC/4801/2021/9). All participants provided informed consent before beginning the study.

##### Materials

As stimuli, we used trait pairs constructed from British perceivers' most frequently spontaneously mentioned trait impressions of highly variable White face images (Sutherland et al., 2018): *friendly, nice, warm, kind, sweet, quiet, funny, shy, young, old, attractive, feminine, masculine* and *intelligent*. These traits were put into 182 unique trait pairs (e.g. *friendly–nice*). These trait pairs were then used to construct sentences that participants saw on their screen together with a 1–7 Likert scale (e.g. *‘If someone is friendly, how likely are they to be nice?’* 1—not at all—4—neutral—7—very).

##### Procedure

The experiment was conducted online through Testable (Rezlescu et al., 2020). Participants were informed that the study was about the relationship between traits. Each participant viewed half (*n* = 91) of the sentences with either one (e.g. *friendly–nice*) or the reverse trait pair presentation (e.g. *nice–friendly*). They rated the similarity between each pair of traits on a 1–7 Likert scale by pressing the keys on their keyboard. The experiment took around 10 min to complete.

##### Conceptual associations data preparation

Similarity ratings of each trait pair were averaged across participants and across presentation versions (e.g. *friendly–nice* averaged with *nice–friendly*), producing 91 average trait pair similarity ratings. We combined *old* and *young* into *age* to achieve one scale by inverting the similarity ratings of pairs including the trait *young* and averaging them with similarity ratings of pairs including the trait *old*. We similarly combined *masculine* and *feminine* into *masculine*. The total of 66 similarity ratings between pairs of traits denoted unique conceptual associations, which were subsequently used in the analyses. For visualization, we collated these ratings into a full 12 x 12 similarity matrix by copying the 66 unique ratings from the lower triangle to the upper triangle symmetrically along the diagonal (see Figure 1b).

#### Face impressions

The face impression data (British perceivers' impressions of White faces) were obtained from a prior study examining British and Chinese perceivers' impressions of highly variable Asian and White face images (Sutherland et al., 2018). In that study, 120 British participants (50% female, *M*_age_ = 20.6 years) judged 500 (50% female) highly variable White faces (Santos & Young, 2005) on traits that were most frequently spontaneously mentioned by British perceivers viewing White faces. The traits were identical to those used in our study to assess conceptual associations, albeit with one scale for *age* (*young* to *old*) and *masculinity (feminine* to *masculine*). In each trial, participants judged one face on one of the traits on a 1–7 Likert scale.

For this study, we obtained the full 12 × 12 matrix of face impressions (symmetrical along the diagonal, see Figure 1d), from which only unique values (e.g. the lower triangle without the diagonal) were used for the analyses. The full 12 × 12 matrix was shown for visualization purposes only.

### Results

#### British conceptual associations cluster along the approachability dimension of White face impressions

We hypothesized that conceptual associations would cluster along the dimensions of approachability, youthful‐attractiveness, and capability, as per Sutherland et al. (2018). We performed a metric multidimensional scaling (MDS) analysis to examine the similarity between the underlying dimensions of conceptual associations and face impressions. Metric MDS, as principal components analysis with varimax rotation (Hout et al., 2013), is primarily used to visualize the positions of individual items (here, traits) on a reduced number of dimensions. As these dimensions are orthogonal, the individual items' positions on each dimension are unaffected by the number of dimensions derived via MDS. Importantly, unlike PCA, MDS only requires a matrix denoting similarities (distances) between individual items (e.g. similarity between traits) rather than ratings of individual items (e.g. ratings of faces on individual traits). This approach is thus useful in our studies, as conceptual associations are collected as relationships or similarities between traits (converted to distances). Given that the main aim of this study is to establish the *similarity* between conceptual associations and face impressions rather than discuss the contents of specific dimensions, here we only briefly outline the similarities between the face and conceptual dimensions (full details in Supporting Information S1).

A three‐dimensional MDS solution explained 79% variation in face impressions (*λ* = 0.88). Dimension one resembled approachability, dimension two resembled youthful‐attractiveness, and dimension three resembled capability (Tables S1 and S2), capturing the dimensions previously derived from the same dataset via PCA (Sutherland et al., 2018). However, interestingly, conceptual associations for the same traits appeared far more complex, with five dimensions needed to approach similar goodness of fit (75%, *λ* = 0.83) to the three‐dimensional face impression solution (Tables S1 and S3). To establish the similarity of the dimensions in face impressions and conceptual associations, we performed a Spearman's rank correlation between the coordinates of face impressions and conceptual associations dimensions. As these specific tests were not pre‐registered, we applied a false discovery rate correction (FDR) to these and all other exploratory tests.

Dimension one in conceptual associations was strongly correlated to dimension one (approachability) of face impressions (*rho*(10) = 0.98, *p* < .0001 both uncorrected and FDR corrected). Dimension two in conceptual associations was strongly related to dimension two in face impressions (youthful‐attractiveness) when uncorrected. However, this correlation did not survive the FDR correction (*rho*(10) = −0.69, *p* < .02 uncorrected, *p* = .13 FDR corrected). No other correlations were significant before or after FDR correction (*ps >* .05). Altogether, conceptual associations appear to reflect approachability (dimension one) of face impressions but not capability and with mixed evidence for youthful‐attractiveness (Figure 1a,c), suggesting that similar but more complex underlying judgements emerge in conceptual associations as compared to impressions of highly variable face images. This solution only partially supported our hypothesis.

#### British conceptual associations positively predict White face impressions

We hypothesized that conceptual associations would predict impressions of highly variable face images via representational similarity analysis (RSA). This analysis establishes the relationship between two (or more) similarity matrices, which denote how similar the same set of items is on two (or more) types of representations. Here, we use RSA to establish how conceptual associations predict face impressions of those same traits. Whilst the MDS showed us which aspects of impression formation are similar between the reduced dimensions of conceptual associations and face impressions, RSA quantifies to what extent these impressions overlap, without reducing the complexity of these multivariate social spaces.

As hypothesized, RSA applied to conceptual associations (Figure 1b) and face impressions (Figure 1d) was significant: conceptual associations positively predicted impressions of highly variable face images (*rho*(64) = .63, *rho* ^2^ = 0.39, *p* < .0002, Figure 1e; see Supporting Information 4.3 for RSAs separated for female vs. male perceivers). Across all studies, *p*‐values for RSAs were estimated using a randomisation test with 5000 iterations (for approach, see Nili et al., 2014). Compared to the original study utilizing impressions of standardized White male faces (Stolier et al., 2018; Study 1 *rho*(76) = 0.82), the relationship between conceptual associations and face impressions found here appeared weaker, but this difference did not survive the FDR correction (*z* = 2.5, *p* = .02, FDR corrected: *p* < .12). Altogether, conceptual associations remain relevant for impressions of highly variable faces with rich visual cues, supporting our hypothesis.

### Discussion

Altogether, our Study 1 findings demonstrate strong parallels between conceptual associations and impressions of highly variable face images. First, approachability is central to conceptual associations and face impressions, albeit dimensions of conceptual trait impressions are more differentiated. Second, conceptual associations positively predict impressions of highly variable face images, sharing 39% of their variance. We found no evidence that the strength of this relationship differed between our study and Stolier et al.'s (2018) prior study with standardized young White male faces (after correction), suggesting that conceptual associations might be as important to impressions derived from highly variable everyday images as they are to impressions of more standardized and constrained face images, despite increased facial cue variation. In sum, our complementary MDS and RSA approaches showed converging support for the dynamic interactive theory, such that abstract conceptual associations share similarities with impressions made to faces, even when the available facial cues are richer.

Thus far, we have addressed our questions by testing British (primarily White) native English speakers who formed impressions of White faces, in line with previous studies' focus on Western cultures. An interesting open question is whether conceptual associations relate to impressions of highly variable faces outside of this cultural context.

## STUDY 2

We set out to understand whether the relationship between conceptual associations and face impressions exists outside of Western perceivers and White faces. First, we hypothesized that Chinese perceivers' conceptual associations would cluster along dimensions previously found to be important for impressions of Asian face images by Chinese perceivers in Mandarin (approachability, youth, attractiveness, and capability: Sutherland et al., 2018). Second, we hypothesized that Chinese perceivers' conceptual associations would predict impressions of highly variable Asian face images, as measured via RSA. Finally, we hypothesized that conceptual associations would predict face impressions of own‐ and other‐culture faces to different extents in both British and Chinese perceivers but left the direction open‐ended. As conceptual associations are shaped by experiences with faces (Stolier et al., 2020) and predict actual regional personality differences (Oh et al., 2022), conceptual associations may predict own‐culture face impressions to a larger extent than other‐culture impressions. Alternatively, conceptual associations may predict own‐culture impressions to a lesser extent than other‐culture impressions, given general other‐culture effects in face processing (e.g. Hayward et al., 2013) and if perceivers rely more on conceptual information to form their judgement. Indeed, conceptual associations may be particularly useful when bottom‐up information is unclear (Freeman et al., 2020). Prior research has shown correspondence between social group‐specific associations and face impressions (Xie et al., 2021), but not whether the same conceptual associations predict impressions of different social groups or cultures to different extents; thus, we left this hypothesis open‐ended. The study was pre‐registered (https://doi.org/10.17605/OSF.IO/GY73D).

### Method

#### Conceptual associations

##### Participants

We recruited 155 participants via Prolific Academic, word of mouth, emails, or the Psychology Research Participation Scheme of the School of Psychology, University of Aberdeen. Participants were compensated £1.75 or partial course credits for their time. Forty‐two participants were excluded in total. Seventeen participants were not fluent or native Chinese (Mandarin/Cantonese) speakers or failed to report their language proficiency, one participant pressed the same key throughout the experiment, and 24 participants failed to answer the attention check correctly.

Our final sample consisted of 113 fluent or native Chinese (Mandarin/Cantonese) speakers (female = 57%, male = 41%, other = 2%), with a mean age of 28 years old (SD ± 7.72 years). Most of our participants were of Asian/Pacific Islander ethnic background (96%), South Asian (2%), or other (2%). All but one participant was a UK resident. The average length of residency was 8.18 years (SD = ±7.49 years). Most participants were nationals of China (54%), the UK (27%), other Asian nationalities (15%) or other world nationalities (4%). The study was approved by the Psychology Ethics Committee of the School of Psychology, University of Aberdeen (PEC/5053/2022/7). All participants provided informed consent before beginning the study.

##### Materials

As experimental stimuli we used trait pairs constructed from the most frequently spontaneously mentioned traits by Chinese participants when viewing highly variable faces in a prior study (Sutherland et al., 2018): *cheerful/outgoing* (开朗), *serious* (严肃), *benevolent* (慈祥), *affable* (和蔼), *kind~and~gentle* (和善), *passionate/enthusiastic* (热情), *capable/experienced* (干练), *diplomatic* (圆滑), *wretched* (猥琐), *attractive* (吸引力), *age* (*young* (年轻人) to *old* (老年人)), and *masculinity* (*feminine* (女性化的) to *masculine* (男性化的)). These traits were put into unique trait pairs (*n* = 91; e.g. *cheerful/outgoing* (开朗)—*serious* (严肃)) and the reverse presentation trait pairs (*n* = 91; e.g. *serious* (严肃)—*cheerful/outgoing* (开朗)). These trait pairs were then used to construct sentences that participants saw on their screen together with a 1–7 Likert scale (e.g. If someone is cheerful/outgoing how likely are they to be serious? (一个开朗的人同时也是一个严肃的人可能性有多大) 1—not at all (完全不可能)—4‐neutral (中立)—7‐very (很可能)). The study was conducted fully in Mandarin and English translations are included here for convenience.

##### Procedure

The instructions, procedure, and duration of the experiment followed Study 1.

##### Conceptual associations data preparation

The data preparation followed Study 1. We collated trait pair ratings including traits *old* and *young* into *age* by inverting the ratings of trait pairs including the trait *young* and averaged them with similarity ratings of pairs including the trait *old*. Similarly, we collated *masculine* and *feminine* into *masculinity*. The 66 similarity ratings denoted unique conceptual associations and were subsequently used in the analyses. These 66 ratings were put into a full 12 × 12 matrix for visualization purposes. Additionally, we used British perceivers' conceptual associations from Study 1 for cross‐cultural comparisons.

#### Face impressions

As in Study 1, the face impression data were obtained from a prior study examining British and Chinese perceivers' impressions of highly variable Asian and White face images (Sutherland et al., 2018). In that study, 120 Chinese participants (50% female, *M*_age_ = 23.6 years) and 120 British participants (50% female, *M*_age_ = 20.6 years) judged 500 (50% female) highly variable Asian (sourced from the internet) or 500 (50% female) highly variable White faces (Santos & Young, 2005) on traits that were most frequently spontaneously mentioned by Chinese perceivers in Mandarin or British perceivers in English. The traits in Mandarin were identical to those used in our Study 2 to assess conceptual associations, albeit with one scale for *age* (*young* to *old*) and one scale for *masculinity* (*feminine* to *masculine*). The English traits are reported in Study 1. Each participant rated the faces on one of the traits on a 1–7 Likert scale.

For this study, we obtained four full 12 × 12 matrices of trait‐by‐trait face impressions: one for Chinese perceivers judging Asian faces, Chinese perceivers judging White faces, British perceivers judging Asian faces, and British perceivers judging White faces (same as Study 1). From each matrix, only unique values (either the lower or the upper triangle without the diagonal) were used for the analyses. The full 12 × 12 matrices were used for visualization purposes only.

### Results

#### Chinese conceptual associations cluster along the approachability, youth, and attractiveness dimensions of Asian face impressions

We hypothesized that conceptual associations would cluster along the dimensions of approachability, youth, attractiveness, and capability, as per Sutherland et al.'s (2018) findings for facial trait impressions. As in Study 1, we performed a metric MDS to examine the similarity of the underlying dimensions of conceptual associations and face impressions (see also Supporting Information S2).

**FIGURE 2:**
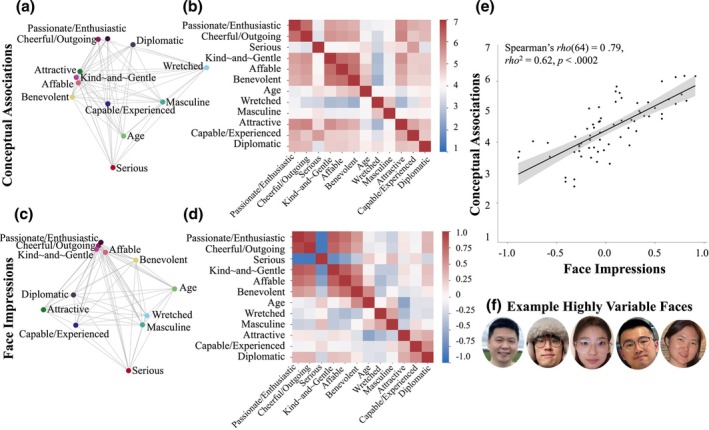
Chinese conceptual associations positively predict Asian face impressions. Multidimensional scaling represents the relationships between traits as rated in conceptual associations (a) and face impressions (c). Each coloured point represents one (the same) trait. Traits (points) that lie closer to each other are also perceived as more similar. Each point in the two‐dimensional MDS plot reflects the position of the trait on the first two dimensions extracted from MDS (approachability and youth for conceptual associations, approachability and attractiveness for face impressions). (b) Conceptual associations matrix (values represent the average similarity between traits, 1 = not at all similar, 7 = very similar). The order of traits is based on their importance to face impressions from prior study of Sutherland et al. (2018). Full matrix is used for visualization and only the values in the lower triangle without the diagonal (unique trait similarities) were used for the analyses. (d) Face impression matrix (values depict Pearson's correlation coefficient). As above, only unique values (lower triangle without the diagonal) were used for analyses. (e) Scatterplot illustrates the relationship between conceptual associations and face impressions based on the lower‐triangle of matrices in b and d used in RSA (raw values, no diagonal). Line of best fit and 95% confidence intervals plotted. (f) Example of Asian highly variable face images varying on cues such as age, gender, pose, lighting, makeup, or accessories.

A four‐dimensional MDS solution explained 84% of variation in face impressions (*λ* = 0.79; Table S4), and dimension one resembled approachability, dimension two attractiveness, dimension three youth, and dimension four capability (Table S5), capturing the original dimensions derived from PCA on the same data (Sutherland et al., 2018). As in Study 1, conceptual associations were more complex: six dimensions were needed to approach the goodness of fit (84%) of the four‐dimensional face impressions MDS model (*λ* = 0.48; Table S6). As in Study 1, we compared the similarity of dimensions via a Spearman's rank correlation with FDR correction applied to non‐pre‐registered analyses. Dimension one in conceptual associations was similar to approachability (dimension one) in face impressions (*rho*(10) = 0.79, *p* < .004 uncorrected, *p* < .05 FDR), so was dimension two in conceptual associations and youth (dimension three) in face impressions (*rho*(10) = −0.83, *p* < .002 uncorrected, *p* < .05 FDR), and dimension three in conceptual associations and attractiveness (dimension two) in face impressions (*rho*(10) = −0.79, *p* < .004, *p* < .05 FDR). No other correlations were significant before or after FDR correction (*ps >* .05). This solution partially supported our hypothesis: conceptual associations reflect the underlying face impression dimensions of approachability, youth, and attractiveness, but not capability (Figure 2a,c), suggesting that similar but more complex underlying judgements emerge in conceptual associations.

#### Chinese conceptual associations positively predict Asian face impressions

As hypothesized, RSA applied to conceptual associations (Figure 2b) and the face impressions (Figure 2d) was significant: Chinese perceivers' conceptual associations positively predicted impressions of highly variable Asian faces (*rho*(64) = 0.79, *rho* ^*2*^ = 0.62, *p* < .0002, Figure 2e; see Supporting Information 4.3 for RSAs separated for female vs. male perceivers). The magnitude was comparable to the original study of conceptual associations and impressions of standardized White male faces by US perceivers in English (Stolier et al., 2018; Study 1 *rho*(76) = 0.82; *z* = 0.43, *p* = .66, FDR corrected *p* > .87). Taken together, results show that there is a positive relationship between conceptual associations and highly variable face impressions outside of the West.

#### Conceptual associations predict impressions similarly across British and Chinese perceivers and Asian and White faces

In addition to using the own‐culture relationships from Study 1 (British conceptual associations and British White face impressions) and Study 2 (Chinese conceptual associations and Chinese Asian face impressions), we also computed the relationships between conceptual associations and other‐culture face impressions. The RSA applied to British conceptual associations and British impressions of Asian faces was significant (*rho*(64) = 0.62, *rho* ^2^ = 0.38, *p* < .0002), as well as the RSA applied to Chinese conceptual associations and Chinese impressions of White faces (*rho*(64) = .76, *rho* ^2^ = .58, *p* < .0002). These results show that conceptual associations predict not only own culture, but also other‐culture face impressions.

We then predicted there would be a difference in the extent to which conceptual associations predict own‐ versus other‐culture face impressions in both British and Chinese perceivers. Unlike our predictions, Fisher's z‐test of difference did not find any evidence that Chinese conceptual associations predicted own‐ and other‐culture face impressions to different extents (*z* = .51, *p* = .61). Similarly, we did not find evidence that British conceptual associations related to own‐ and other‐culture face impressions to different extents (*z* = .05, *p* = .96). Thus, we found no evidence of own or other‐culture effects in the relationship between conceptual associations and face impressions in Chinese or British perceivers.

Finally, we tested whether British and Chinese perceivers differ in the extent to which their conceptual associations predict face impressions more generally. Exploratory Fisher's *z*‐tests of difference initially showed that Chinese perceivers' conceptual associations related to Asian face impressions marginally more than British perceivers' conceptual associations related to White face impressions; however, this difference did not survive the FDR correction (*z* = 1.98, *p* < .05, FDR corrected *p* = .29). Initially, it appeared that Chinese conceptual associations predicted Asian face impressions to a larger extent than British conceptual associations; again, this difference did not survive the FDR correction (*z* = 2.04, *p* < .05, FDR corrected *p* = .29). We found no evidence to suggest that Chinese or British perceivers' conceptual associations differed in the extent to which they predicted other‐culture face impressions (*z* = 1.53, *p* = .13, FDR corrected *p* = .62) and White face impressions (*z* = 1.48, *p* = .14, FDR corrected *p* = .62). Thus, we found no strong evidence to suggest that Chinese and British perceivers differ in the extent to which their conceptual associations predict face impressions.

### Discussion

Overall, in Study 2, we demonstrated that conceptual associations show strong correspondence with impressions of highly variable face images across cultures. First, approachability, youth, and attractiveness underlie Chinese conceptual associations in Mandarin, as well as their impressions of highly variable Asian faces. Second, and critically, we found a strong positive relationship between Chinese perceivers' conceptual associations and Asian face impressions, sharing 62% variance. The strength of this relationship was not different from that of the original study of conceptual associations (Stolier et al., 2018). Third, we did not find evidence for any own or other‐culture effects in this relationship or any differences between Chinese and British perceivers in the extent to which conceptual associations predicted face impressions. Altogether, this study provides cross‐cultural evidence supporting dynamic interactive theory for the first time.

One limitation of Studies 1 and 2 findings thus far is that both were obtained from average data from separate participant samples providing face impressions and conceptual associations. Although this approach is widely used in first impressions and provides an important first test of the idea that conceptual associations can predict impressions of faces, it does not address this relationship in individual participants. In Study 3, we therefore sought to assess whether individual differences in participants' conceptual associations would predict individual differences in impressions of highly variable faces.

## STUDY 3

Here, we provide a stricter final test for the dynamic interactive theory by examining the relationship between conceptual associations and highly variable face impressions across individual perceivers. In this study, the same participants made impressions of faces and were then asked for their conceptual associations. Participants served as the unit of analysis, with one score for their conceptual associations and one score for the face trait judgement similarity. Critically, we hypothesized that participants' idiosyncratic conceptual associations would correlate with their face impressions, as measured via Spearman's rank correlation. We also aimed to cross‐validate our previous findings by again testing whether group conceptual associations would predict group face impressions, averaged across participants, and compare the strength of this relationship to Studies 1 and 2. The study was pre‐registered (https://doi.org/10.17605/OSF.IO/XRWE4).

### Method (face impressions and conceptual associations)

#### Participants

The final sample was 244 participants, 15–17 participants per trait pair (female = 48%, male = 50%, other = 2%; *M*_age_ = 35.52 years old, SD ± 11.89). Most participants were White (84%), followed by Asian (10%), Black (4%), or other or non‐specified ethnicity (2%). Most participants were UK nationals (80%), other European nationality (9%), other world nationality (8%) or non‐specified (3%). All participants were either native or fluent English speakers.

Participants were recruited from Testable Minds and were paid $2.50 for their time. We recruited 254 participants (sample size based on Stolier et al., 2018, Study 2). Ten participants were excluded in total. Six participants pressed the same key throughout at least one block of trials, one experienced technical issues, and three failed to correctly answer the attention check. The study was approved by the Psychology Ethics Committee of the School of Psychology, University of Aberdeen (PEC/5057/2022/8). All participants provided informed consent before beginning the study.

#### Materials

##### Face stimuli

The highly variable face images were taken from the US10k Database (Bainbridge et al., 2013). We randomly chose a subset of 100 White forward‐facing, non‐famous, and high‐quality face images (50 female, 50 male).

##### Trait stimuli

The chosen traits were a subset of those from Study 1. We chose six traits, two to represent each dimension of face impressions: *friendly* and *nice* (approachability), *attractive* and *masculine* (youthful‐attractiveness), and *old* and *intelligent* (capability). These six traits were put into all unique pairwise combinations of trait pairs, yielding 15 trait pair conditions, including across dimensions, for example attractive‐intelligent, given our focus on multivariate impressions.

#### Procedure

The experiment was administered online through Testable (Rezlescu et al., 2020). Participants were randomly assigned to one of the 15 trait pairs (conditions). All participants first completed the face impression task and then the conceptual associations task to minimize demand bias. In the face impressions task, participants first rated the full set of face images on one trait (trait block 1), then the same set on a second trait (trait block 2) using a 1–7 Likert scale (e.g. *‘How attractive is this person?’* 1–not at all, 7–very). The order of face images was randomized within each trait block, as was the order of trait blocks. In the subsequent conceptual associations task, they rated the conceptual associations between the two traits in this format: for example *‘If someone is attractive, how likely are they to be friendly?’* and *‘If someone is friendly, how likely are they to be attractive?’*. The order of conceptual trait association questions was randomized.

#### Data preparation

Given the small set of traits used, the underlying dimensions of conceptual associations and face impressions were not examined here. To assess the relationship between individuals' own conceptual associations and their idiosyncratic face impressions, we obtained two scores per participant: their conceptual belief score (average of responses to the two questions in the conceptual task, that is how similar they judged these traits to be) and their face impression score (Pearson's correlation coefficient between face ratings on the first trait and the second trait, i.e. how similar they judged faces on these two traits). For example, a participant assigned to judge faces on attractiveness and friendliness would have one conceptual belief score and one face impression score for the similarity between attractiveness and friendliness. These scores were used in subsequent individual differences analysis.

**FIGURE 3:**
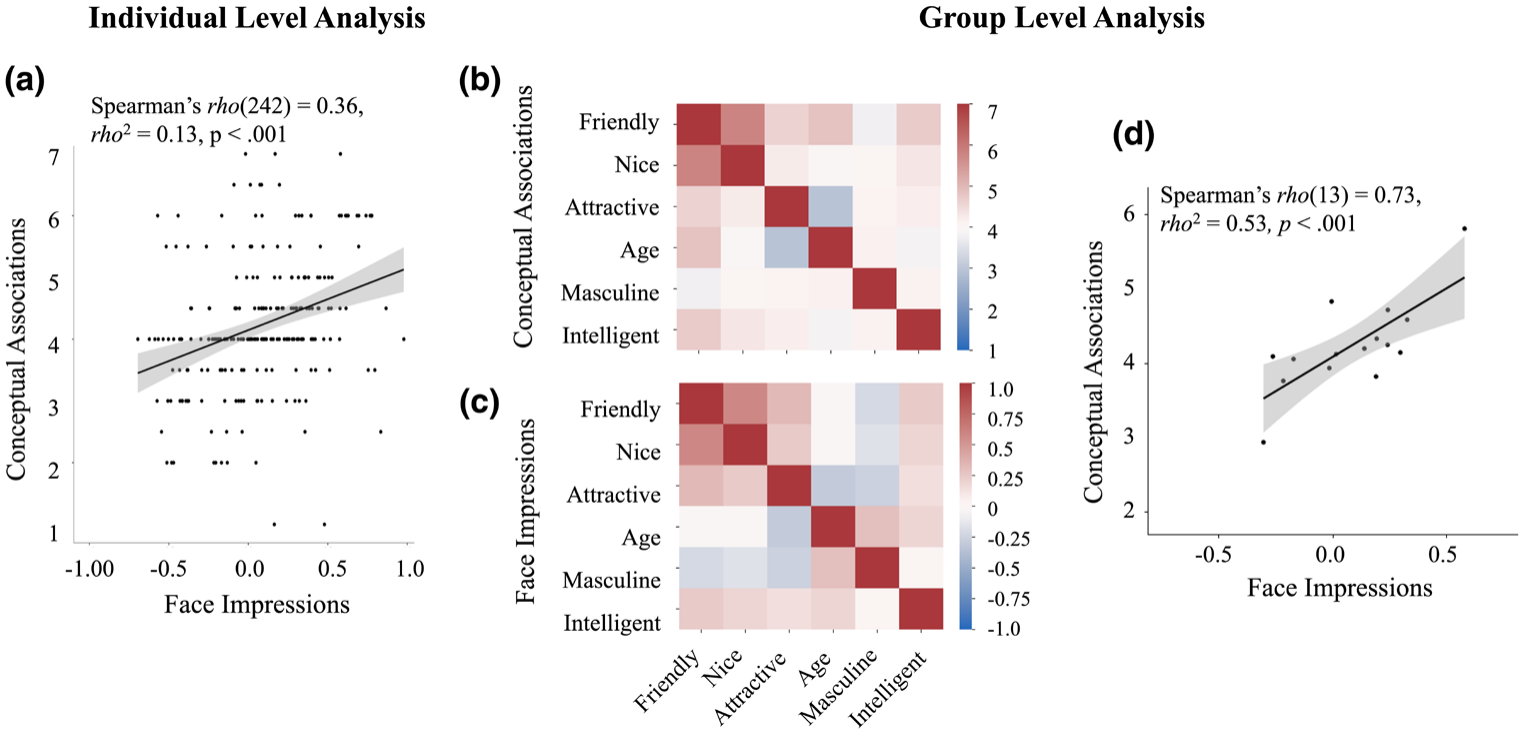
Individual and group level conceptual associations positively relate to individual and group level face impressions. (a) Scatterplot illustrates the relationship between individual conceptual associations and face impressions. Each point represents one participant and one pair of traits. Line of best fit and 95% confidence intervals are plotted. (b) The conceptual associations matrix represents the similarity between traits (averaged across participants, 1 = not at all similar, 7 = very similar). Traits were ordered based on the importance of dimensions from Sutherland et al. (2018) to which they were related: Friendly and nice for approachability, attractive and age for youthful‐attractiveness, and masculine and intelligent for capability. Full matrix for visualization only, and only the values in the lower triangle without the diagonal (unique trait similarities) were used for the analyses. (c) The face impressions matrix represents the similarity between traits (averaged across participants, Pearson's correlation coefficient). Only unique values (lower triangle without the diagonal) were used for analyses. (d) Scatterplot shows the relationship between group conceptual associations and group face impressions. Each point represents a trait pair. Line of best fit and 95% confidence intervals are plotted.

To analyse the group level data (as a replication of Study 1 and 2), these scores were later grouped into a conceptual associations matrix (averages of individuals' conceptual associations per trait pair) and a face impression matrix (average of individuals' face impressions per trait). As in Study 1 and 2, only the 15 unique trait pairs were used in the statistical analysis of the group RSAs (lower part of full matrix without the diagonal), and the full matrices are shown only for visualization (Figure 3b,c).

### Results

#### Individual participants' conceptual associations positively relate to their idiosyncratic face impressions

We hypothesized that individual participants' conceptual associations would correlate positively with individual participants' face impressions. Spearman's rank correlation applied to individual participants' conceptual associations and face impressions showed that there indeed was a positive relationship between the two, *rho*(242) = 0.36, *rho* ^2^ = 0.13, *p* < .001 (Figure 3a; see Supporting Information 4.3 and Figure S3 for analysis separated for female vs. male perceivers and faces), supporting our hypothesis. An exploratory analysis did not show differences in the extent to which individuals' conceptual associations predicted idiosyncratic impressions of highly variable faces in our study versus the original study utilizing standardized young White male faces (Stolier et al., 2018, Study 2 *rho*(204) = 0.34; *z* = 0.24, *p* = .81, FDR corrected *p* = .88). This finding suggests that individuals with more similar conceptual associations will also judge highly variable faces more similarly on those traits.

#### Group level conceptual associations positively predict group level face impressions

We also hypothesized that there would be a positive relationship between face impressions and conceptual associations when averaged across the group. Indeed, replicating Studies 1 and 2, RSA applied to conceptual associations (Figure 3b) and face impressions (Figure 3c) was significant: *rho*(13) = .73, *rho* ^2^ = .53, *p* < .001 (Figure 3d). An exploratory analysis did not show differences in the extent to which group conceptual associations predicted group face impressions in Study 3 and the original conceptual associations study (Stolier et al., 2018 Study 1; *z* = 0.74, *p* = .46, FDR corrected *p* > .87), the present Study 1 and Study 3 (*z* = 0.62, *p* = .53, FDR corrected *p* > .87), and the present Study 2 and Study 3 (*z* = 0.50, *p* = .62, FDR corrected *p* > .87). Together, these results show that there is a positive relationship between the group‐level conceptual associations and highly variable face impressions, across three independent groups of participants and three independent face databases.

### Discussion

Together, these findings show that individuals' conceptual associations are positively related to individuals' own impressions of highly variable images (sharing 13% variance), suggesting that conceptual associations could represent a source of individual variation in impressions of highly variable faces. We also replicated our Study 1 and 2 findings, showing that group conceptual associations consistently predict group impressions of highly variable face images, with a third group of participants and new face images. This relationship was similarly strong across our three studies and the original study of conceptual associations, showcasing the robustness of the relationship between conceptual associations and face impressions.

## MAIN DISCUSSION

The dynamic interactive theory suggests that visual cues combine with conceptual associations about traits to result in impressions. Across three studies, we demonstrated that even with an increased richness of visual cues, conceptual associations positively predict face impressions in groups of perceivers (Study 1), across cultures (Study 2), and across individuals (Study 3). Our findings are in line with the dynamic interactive theory (Freeman et al., 2020). We note that while our current findings are supportive of this theory, other studies of more variable, naturalistic, or non‐White male faces have found that such faces can lead to richer face impressions than standardized face images (e.g. Sutherland et al., 2013, 2018). Here we show that, despite this increased richness in facial cues, conceptual impressions shared considerable variance with resulting impressions of these faces. Remarkably, 39% and 62% of the variance was shared between face impressions and conceptual associations at the group level, and 13% at the individual perceiver level.

Our results show high agreement with previous work with standardized young White faces (e.g. Stolier et al., 2018, 2020). There was no difference in the extent to which conceptual associations predicted face impressions across our three studies, or between our studies and the original studies of Stolier et al. (2018). Further, in support of the relationship between conceptual associations and face impressions, we found that perceivers' conceptual associations and highly variable face impressions shared (some) common underlying dimensions. Interestingly, conceptual associations were more highly dimensional than face impressions, suggesting that the understanding of traits is more nuanced in conceptual evaluations, whereas certain visual cues might be especially salient or constraining of face judgements. Interestingly, the higher dimensionality of conceptual associations compared to actual perceptual judgements has also previously been noted for romantic partner evaluations (South Palomares & Young, 2019). Here, perceivers conceptually indicated that many factors influence partner preferences, yet the actual face‐based partner preferences were primarily driven by only one of those factors (South Palomares & Young, 2019).

We also find evidence for the importance of conceptual associations outside of Western cultures. Chinese perceivers' conceptual associations positively predicted impressions of highly variable own‐culture faces to a similar extent as British (Study 1 & 3) and US perceivers (Stolier et al., 2018). Furthermore, Chinese perceivers' conceptual associations share common underlying dimensions with impressions of highly variable Asian faces, and like the underlying components of British perceivers' conceptual associations, they are also more differentiated than face impressions. Interestingly, we found no indication that British and Chinese perceivers differ in the extent to which their conceptual associations predict impressions of highly variable everyday Asian and White face images. Our findings thus open the possibility that conceptual associations may be applied to impressions of different social groups to infer judgements. Prior research has shown that specific stereotypical conceptual associations (e.g. ‘How likely is a dominant man to be strong?’) predicted impressions of related social groups (Xie et al., 2021), but it is not currently well understood how specific stereotypes interact with other, perhaps more general conceptual associations. For example, if one judges men to be ‘strong’ and ‘dominant’, it is unclear if this association is inferred via a specific gender stereotype (men are both strong and dominant) or via a more general association (strong people are dominant), resulting in an impression that is congruent with both the stereotype and the general beliefs. Future more detailed studies can fully elucidate the specific cognitive or other mechanisms through which conceptual associations (whether stereotypical or more general) inform impressions.

Finally, we found that face impressions and conceptual associations correspond across individuals, such that the more an individual believes two traits to be similar conceptually, the more they judge faces as similar on those traits (Study 3; see also Stolier et al., 2018). Our findings suggest that the richness of visual cues does not necessarily change the strength of the relationship between conceptual associations and face impressions in individuals' impressions. Interestingly, individual differences in face impressions are suggested to be stronger when images are highly variable (Hehman et al., 2017), yet conceptual associations are argued to be less influential in instances when images provide more information (Freeman et al., 2020). These contrasting strands, together with the similarity of our findings and those of Stolier et al. (2018), call for further examination of the mechanisms through which conceptual associations relate to impressions at different degrees of face variability at the individual perceiver level. For instance, future research may wish to systematically manipulate face variability and/or different individual conceptual associations, perhaps in combination.

Taken together, our findings strongly support the relationship between conceptual associations and impressions of highly variable faces in line with the dynamic interactive theory. Unlike prior studies, we further advance the understanding of this relationship by showing which underlying dimensions are shared by face impressions and conceptual associations. We thus establish a crucial step to understanding how conceptual associations are derived from and applied to impressions in everyday life. More generally, a correspondence between perceptual and conceptual representations can be found in many areas of psychology, including object categorization (Lupyan et al., 2010), colour categorization (Mitterer et al., 2009; Witzel et al., 2011), and emotion perception (Young & Bruce, 2023). Kuhn et al. (2017) showed that emotions rated in faces corresponded to emotions from voices above and beyond their low‐level visual and auditory similarities, suggesting a common conceptual structure underlying emotion perception across modalities. Later work of Brooks and Freeman (2018) showed a more direct relationship between conceptual and perceptual representations of emotion, finding that conceptual emotion associations (e.g. how likely are angry people to be sad?) also predicted how similarly emotions were perceived in faces. Our findings thus not only add to the trait impression literature, but also to the wider body of work linking perceptual and conceptual representations.

We acknowledge the limitations of the current work. First, we deliberately do not make strong inferences about the causality of the relationship between conceptual associations and highly variable face impressions. Indeed, we think the relationship is likely to be bidirectional, such that impressions from beliefs and facial cues mutually reinforce each other. One paper has recently tested causal links, showing that conceptual associations can indeed causally influence impressions of standardized young White male faces, as well as vice versa (Stolier et al., 2020). Our current studies suggest that future work may fruitfully use more highly variable faces to test the relative strength of causal relationships going forward. Second, as we were focused on understanding trait impressions at a multivariate level, our methodology did not disentangle how individual impressions (e.g. trustworthiness) may be informed by conceptual associations. Whilst we address which dimensions are similar across the two spaces using MDS, some individual traits may be more readily derived from facial visual cues and then inform other traits via conceptual associations, whilst others may be more dependent on these prior conceptual associations. Future work may wish to further disentangle the dynamics of individual trait impression formation within these complex spaces.

Whilst we extend the ecological validity of the dynamic interactive theory, our findings are still based on impressions of static faces. There are important situations in which we might make impressions just from such faces (e.g. online, from CVs, etc.); however, people can form impressions from a wide variety of cues, not only visual but also auditory (Lavan, 2023), olfactory (Groyecka et al., 2017), or from movement dynamics (Fink et al., 2015). Moreover, individual differences also exist in these impressions (for example, as recently shown for voices: Lavan & Sutherland, 2024). To further extend our understanding of real‐life impression formation, it is crucial to consider how these external cues integrate not only with each other but also with perceivers' pre‐existing conceptual associations to inform multi‐modal impressions. Likewise, we tested only two cultures here, but future work may wish to extend the cross‐cultural comparisons. Our current work suggests interesting future avenues will be to test whether underlying conceptual associations can explain impressions from a diverse range of possible cues.

## CONCLUSION

Our findings extend the current understanding of the role of the perceiver in face impressions in line with the dynamic interactive theory of person construal. We find that conceptual associations predict impressions of highly variable faces, across British and Chinese cultural groups, and within individuals. Together, our findings showcase the importance of the perceiver in impressions of everyday face images across cultures, and more broadly, the links between abstract concepts and face perceptions.

## AUTHOR CONTRIBUTIONS

**Barbora Illithova:** Conceptualization; methodology; formal analysis; writing – original draft. **Andrew W. Young:** Conceptualization; writing – review and editing. **Mingyuan Chu:** Writing – review and editing; resources. **Clare A. M. Sutherland:** Conceptualization; supervision; writing – review and editing.

## CONFLICT OF INTEREST STATEMENT

No competing interests to declare.

## Supporting information


Data S1.

## Data Availability

All hypotheses, methods, and most analyses were pre‐registered. Additional analyses or analytical decisions that were not pre‐registered are labelled as exploratory, and a false discovery rate correction for multiple comparisons is applied to all such analyses. Raw data, full analysis scripts, software versions, and links to pre‐registrations are available on Open Science Framework (https://osf.io/bjz8n/?view_only=6308fcf0338340b9b336a6256b38ea0c), as are pre‐registrations for Study 1 (https://doi.org/10.17605/OSF.IO/X453B), Study 2 (https://doi.org/10.17605/OSF.IO/GY73D), and Study 3 (https://doi.org/10.17605/OSF.IO/XRWE4).
