# Supplementary material for: Conceptual trait associations predict impressions of highly variable faces
Source: Br J Psychol. 2025 Sep 24;117(1):337–55. doi: 10.1111/bjop.70031 (PMC12783882; doi:10.1111/bjop.70031)
Supplement: Supplementary file 1 — Data S1. [file BJOP-117-337-s001.docx]

**Supplementary Materials 1**

**1. Study 1**

*1.1 Face Impressions & Conceptual Trait Associations Multidimensional Scaling*

*1.1.1 Data Transformations*

Multidimensional scaling (MDS) requires a distance matrix. The face impressions correlation matrix was transformed into a Euclidean distance matrix using the following formula: (*d* = $\sqrt{2\cdot\left( 1-r \right)}$), where: d= Euclidean distance and r = correlation coefficient. This face impressions Euclidean distance matrix was then used in the MDS.

For comparability in scale with the face impressions, we first transformed the conceptual trait associations similarity matrix (ranging from 1 = very dissimilar, to 7= very similar) into a quasi-correlation matrix, where 1 (very dissimilar) was scaled to –1, and 7 (very similar) to 1, with 4 (neutral) equaling 0. This quasi-correlation matrix was then transformed into a Euclidean distance matrix following the same formula as the face impression matrix. We then applied the MDS to the conceptual trait associations Euclidean distance matrix.

- - 1. *Dimension Reduction*

Based on the eigenvalues, and the goodness of fit measures (GOF 1 and GOF 2, see Note **Table S1**), it appears that a two to three-dimensional solution to face impressions is appropriate (see **Table S1**). Interestingly, conceptual trait associations appear more highly dimensional than face impressions: five to six dimensions are needed to achieve a similar goodness of fit to the three dimensions of face impressions (**Table S1**).

**Table S1**

*Goodness of Fit and Eigenvalues per Number of Axes in British Face Impressions MDS and Conceptual Trait Associations MDS*

| Number of Axes | *British Face Impressions MDS* | | | *British Conceptual Trait Associations MDS* | |
| --- | --- | --- | --- | --- | --- |
| Number of axes (k) | GOF 1 | GOF 2 | Eigenvalues ($\lambda$) | GOF | Eigenvalues ($\lambda$) |
| 1 | 0.48 | 0.48 | 4.76 | 0.23 | 2.19 |
| 2 | 0.70 | 0.70 | 2.22 | 0.43 | 1.90 |
| 3 | **0.79** | **0.79** | 0.88 | 0.56 | 1.25 |
| 4 | 0.88 | 0.88 | 0.84 | 0.66 | 0.99 |
| 5 | 0.91 | 0.91 | 0.33 | **0.75** | 0.83 |
| 6 | 0.94 | 0.94 | 0.30 | 0.82 | 0.66 |
| 7 | 0.96 | 0.96 | 0.18 | 0.86 | 0.47 |
| 8 | 0.97 | 0.97 | 0.13 | 0.91 | 0.39 |
| 9 | 0.98 | 0.98 | 0.11 | 0.94 | 0.35 |
| 10 | 0.99 | 0.99 | 0.09 | 0.97 | 0.29 |
| 11 | <1.00 | 1.00 | 0.07 | 1.00 | 0.26 |
| 12 | - | - | -0.01 | - | < 0.01 |

*Note*. Goodness of fit measure 1 (GOF 1) is expressed as $\left( \sum_{j=1}^{k} \lambda_{j} \right)\div\left( \sum_{j=1}^{n} \left| \lambda_{j} \right| \right)$. Goodness of fit measure 2 (GOF 2) is expressed as $\left( \sum_{j=1}^{k} \lambda_{j} \right)\div\left( \sum_{j=1}^{n} \lambda_{j} \right); \lambda_{j}= the jth eigenvalue; \lambda_{j} \in\left( 0 , \infty\right)$ ; k = number of axes, k $\in$<1, n-1>; n = total number of elements (traits). When all eigenvalues are positive, the GOF 1 and GOF 2 measures are equal, denoted as GOF.

- - 1. *Dimension Conceptualization*

The three-dimensional face impression MDS solution (**Table S2**) (unsurprisingly) approximates the three dimensions as identified via PCA on the same dataset: approachability, youthful-attractiveness, and capability. Traits like friendly, nice, warm, kind, sweet, quiet and shy load strongly onto Axis 1, as in the original approachability dimension in Sutherland et al. (2018). Traits like attractive, age, masculine and funny load highly onto Axis 2, similar to the youthful-attractiveness dimension in Sutherland et al. (2018). Axis 3 is described by intelligent, funny, and shy, similarly to the capability dimension.

**Table S2**

*Coordinates for Each of the 12 Traits for the First Three Axes Describing British Face Impressions*

| Item (Trait) | Axis | | |
| --- | --- | --- | --- |
|  | 1 | 2 | 3 |
| Friendly | **-0.61** | -0.03 | -0.11 |
| Nice | **-0.60** | 0.09 | -0.02 |
| Warm | **-0.60** | 0.04 | -0.04 |
| Kind | **-0.57** | <0.01 | -0.07 |
| Sweet | **-0.59** | 0.16 | -0.04 |
| Quiet | **1.18** | **0.42** | 0.02 |
| Shy | **0.96** | 0.21 | **-0.44** |
| Funny | **-0.30** | **-0.48** | **-0.33** |
| Attractive | -0.05 | **0.95** | 0.18 |
| Age | **0.35** | -**0.72** | 0.18 |
| Masculine | **0.67** | **-0.52** | -0.04 |
| Intelligent | 0.14 | -0.18 | **0.70** |

*Note.* Coordinates above |0.3| are in bold.

Conceptual trait associations are more dimensional but show similarities to face impressions (**Table S3**). For example, in Axes 1, friendly, nice, warm, kind, and sweet lie opposite of quiet, shy, age, and masculine. In Axes 2, age, masculine and funny lie opposite of quiet. The order of traits is based on a prior study, which identified these traits as the most common spontaneously mentioned impressions (Sutherland et al., 2018).

**Table S3**

*Coordinates for Each of the 12 Traits for the First Five Axes Describing British Conceptual Trait Associations*

| Item (Trait) | Axis | | | | |
| --- | --- | --- | --- | --- | --- |
|  | 1 | 2 | 3 | 4 | 5 |
| Friendly | **-0.54** | -0.05 | -0.08 | -0.03 | -0.14 |
| Nice | **-0.44** | 0.20 | -0.08 | -0.09 | -0.10 |
| Warm | **-0.51** | 0.06 | -0.11 | -0.01 | 0.02 |
| Kind | **-0.41** | 0.23 | -0.13 | -0.07 | -0.05 |
| Sweet | **-0.32** | **0.34** | -0.02 | -0.04 | 0.12 |
| Quiet | **0.59** | **0.58** | -0.01 | -0.04 | 0.01 |
| Shy | **0.57** | **0.63** | 0.15 | -0.06 | -0.11 |
| Funny | -0.11 | **-0.63** | 0.21 | 0.20 | -0.27 |
| Attractive | -0.01 | -0.25 | **0.76** | -0.06 | **0.56** |
| Age | **0.32** | **-0.30** | **-0.70** | **0.34** | **0.48** |
| Masculine | **0.49** | **-0.61** | -0.18 | **-0.70** | -0.15 |
| Intelligent | **0.37** | -0.19 | 0.18 | **0.56** | **-0.37** |

*Note.* Coordinates above |0.3| are shown in bold. The order of traits is based on a prior study, which identified these traits as the most common spontaneously mentioned impressions (Sutherland et al., 2018).

**Supplementary Materials 2**

**2.Study 2**

*2.1 Face Impressions & Conceptual Trait Associations Multidimensional Scaling*

*2.1.1 Data Transformation*

The face impression correlation matrix and conceptual trait associations similarity matrix were both transformed into Euclidean distance matrices as in Study 1.

*2.1.2 Dimension Reduction*

Based on the eigenvalues and the goodness of fit measure (GOF), it appears that a three to four-dimensional solution to Chinese face impressions is appropriate (again, unsurprisingly, given the original dataset; see **Table S4**). Interestingly, however, and in agreement with Study 1, the Chinese conceptual trait associations appear more highly dimensional than the face impressions do. Five to six dimensions are needed to achieve a similar goodness of fit to the three-to-four-dimensional solution of face impressions (**Table S4**).

**Table S4**

*Goodness of Fit Measure per Number of Axes in Chinese Face Impressions MDS and Conceptual Trait Associations MDS*

| Number of Axes | *Chinese Face Impressions MDS* | | *Chinese Conceptual Trait Associations MDS* | |
| --- | --- | --- | --- | --- |
|  | GOF | Eigenvalues ($\lambda$) | GOF | Eigenvalues ($\lambda$) |
| 1 | 0.40 | 4.21 | 0.29 | 2.74 |
| 2 | 0.63 | 2.33 | 0.47 | 1.67 |
| 3 | 0.76 | 1.46 | 0.63 | 1.43 |
| 4 | **0.84** | 0.79 | 0.73 | 0.90 |
| 5 | 0.89 | 0.51 | 0.79 | 0.63 |
| 6 | 0.92 | 0.35 | **0.84** | 0.48 |
| 7 | 0.95 | 0.27 | 0.89 | 0.43 |
| 8 | 0.97 | 0.19 | 0.92 | 0.31 |
| 9 | 0.98 | 0.15 | 0.95 | 0.25 |
| 10 | 0.99 | 0.13 | 0.98 | 0.23 |
| 11 | 1.00 | 0.08 | 1.00 | 0.21 |
| 12 | - | -6.654500e-17 | - | 2.957393e-16 |

- - 1. *Dimension Conceptualization*

The first four axes of the Chinese face impressions MDS approximate the first four dimensions identified on the same data via PCA in the original study (Sutherland et al., 2018). Axis 1 appears to reflect approachability, Axis 2 appears to reflect attractiveness, Axis 3 youth, and Axis 4 capability.

**Table S5**

*Coordinates for Each of the 12 Traits for the First Four Axes of Chinese Face Impressions*

| Item (Trait) | Axis | | | |
| --- | --- | --- | --- | --- |
|  | 1 | 2 | 3 | 4 |
| Passionate/Enthusiastic | **-0.70** | 0.06 | 0.24 | <. 0.01 |
| Cheerful/Outgoing | **-0.67** | 0.03 | **0.34** | 0.01 |
| Serious | **1.07** | -0.05 | **-0.38** | -0.28 |
| Kind-and-Gentle | **-0.69** | 0.06 | 0.01 | -0.11 |
| Affable | **-0.61** | 0.19 | -0.24 | -0.09 |
| Benevolent | **-0.31** | **0.52** | **-0.40** | 0.02 |
| Age | **0.36** | **0.73** | **-0.37** | 0.13 |
| Wretched | **0.61** | 0.25 | **0.82** | -0.13 |
| Masculinity | **0.73** | 0.21 | 0.29 | 0.11 |
| Attractive | -0.04 | **-0.81** | -0.14 | **-0.52** |
| Capable/Experienced | **0.35** | **-0.63** | -0.14 | **0.43** |
| Diplomatic | -0.10 | **-0.56** | -0.02 | **0.43** |

*Note.* Coordinates above |0.3| are shown in bold. The order of traits is based on a prior study, which identified these traits as the most common spontaneously mentioned impressions (Sutherland et al., 2018).

Conceptual trait associations are more dimensional but show similarities to face impressions (**Table S6**). Like in face impressions Axis 1, traits cheerful/outgoing, kind-and-gentle, affable, and benevolent lie opposite of traits like serious, age, wretched, and masculinity on conceptual trait associations Axis 1. Similarly, in face impression Axis 2 and conceptual trait associations Axis 3, attractive and capable/experienced lie opposite of age. Axis 3 in face impressions and Axis 2 in conceptual trait associations cheerful/outgoing and wretched lie opposite of serious and age. In Axes 4, capable/experienced and diplomatic lie closely together.

**Table S6**

*Coordinates for Each of the 12 Traits for the First Six Axes of Chinese Conceptual Trait Associations*

| Item (Trait) | Axis | | | | | |
| --- | --- | --- | --- | --- | --- | --- |
|  | 1 | 2 | 3 | 4 | 5 | 6 |
| Passionate/Enthusiastic | -0.28 | **-0.44** | 0.17 | -0.05 | **0.35** | 0.01 |
| Cheerful/Outgoing | **-0.39** | **-0.41** | 0.07 | 0.10 | 0.25 | 0.26 |
| Serious | **0.35** | **0.73** | **0.47** | -0.04 | 0.05 | -0.09 |
| Kind-and-Gentle | **-0.51** | 0.06 | -0.17 | 0.16 | -0.08 | **-0.30** |
| Affable | **-0.46** | 0.13 | **-0.31** | 0.11 | -0.08 | -0.24 |
| Benevolent | **-0.45** | 0.26 | **-0.34** | 0.13 | -0.05 | -0.01 |
| Age | **0.31** | **0.50** | **-0.63** | -0.24 | 0.21 | **0.31** |
| Wretched | **0.98** | **-0.46** | -0.20 | -0.08 | 0.20 | **-0.31** |
| Masculinity | **0.69** | -0.07 | 0.10 | **0.67** | -0.23 | 0.18 |
| Attractive | **-0.42** | -0.10 | **0.41** | 0.04 | -0.08 | 0.14 |
| Capable/Experienced | 0.05 | 0.21 | **0.56** | **-0.32** | 0.01 | -0.06 |
| Diplomatic | 0.11 | **-0.41** | -0.12 | **-0.47** | **-0.53** | 0.10 |

*Note.* Coordinates above |0.3| are shown in bold. The order of traits is based on a prior study, which identified these traits as the most common spontaneously mentioned impressions (Sutherland et al., 2018).

**Supplementary Materials 3**

**3.Study 3**

*3.1. Supplementary Results to Study 3*

*3.1.1 Conceptual Trait Associations and Face Impressions are Reliable*

We hypothesised that there would be a positive relationship between the group-level conceptual trait associations from Study 1 (**Fig S1A**) and Study 3 (**Fig S1B**). Note, we looked specifically at Studies 1 and 3 as these were both tested in the same language. It was not possible to directly compare Study 2, as these conceptual trait associations represented Chinese concepts i.e. in a different language. As predicted, the representational similarity analysis applied to the conceptual trait associations was significant: Spearman’s *rho*(13) = 0.70, *rho*^2^ = 0.49, *p* < .003 (**Fig S1C**). Similarly, we hypothesized that there would be a positive relationship between the group-level face impressions from Study 1 (**Fig S1D**) and Study 3 (**Fig S1E**). The representational similarity analysis applied to the face impression matrices was significant: Spearman’s *rho* (13) = 0.88, *rho*^2^ = 0.77, *p* < .0002 (**Fig S1F**). Thus, conceptual trait associations are highly similar between two independent groups of British perceivers tested six months apart. Likewise, face impressions are highly similar between two independent groups of British perceivers: one tested in-person, and the second one online, using two different databases of highly variable face images. This finding suggests that conceptual trait associations and face impressions are reliable across groups of British perceivers.

**Figure S1**

*British Conceptual Trait Associations and British Face Impressions are Socially Shared
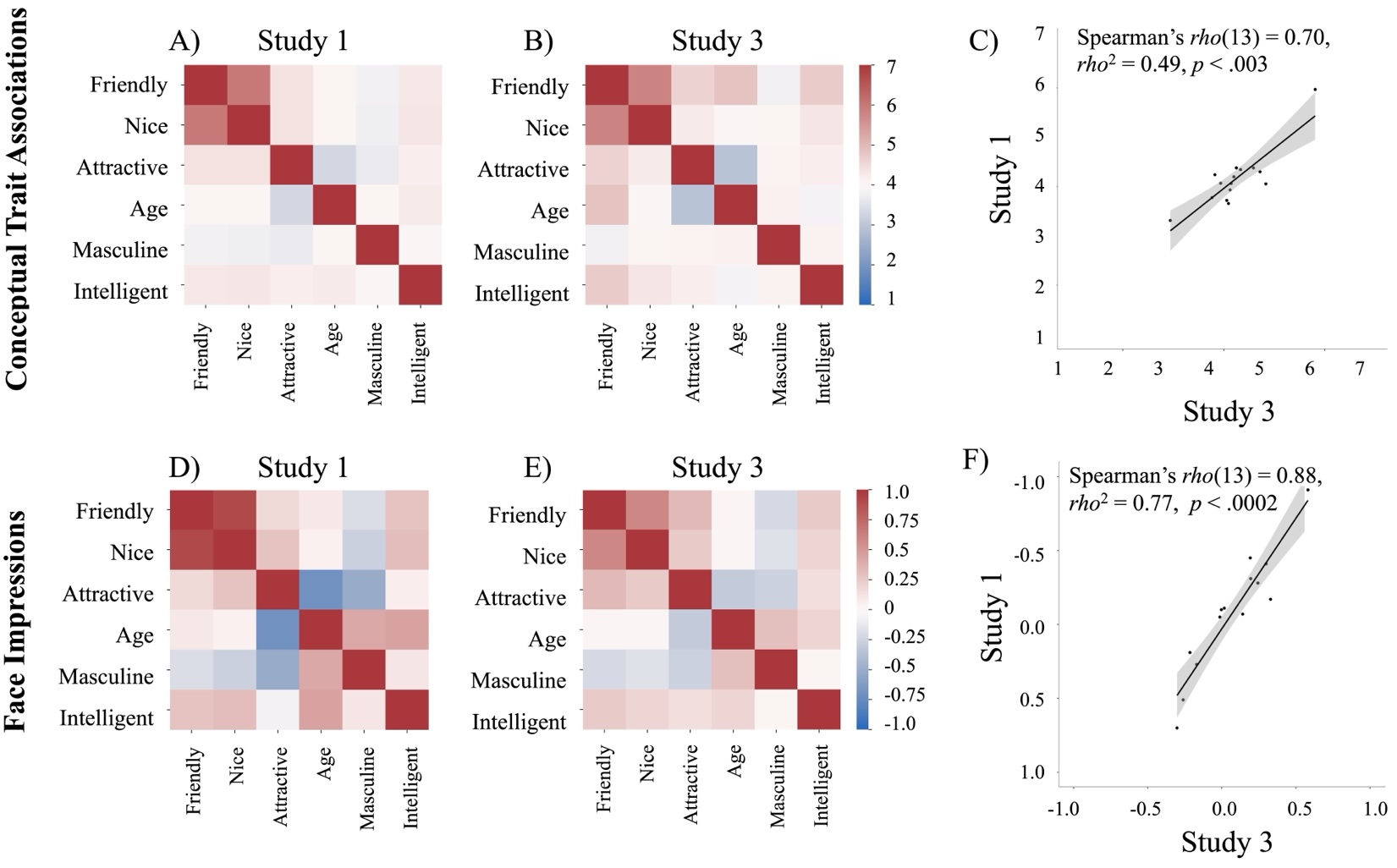

Note.* **Conceptual Trait Associations**: A) Subset of Study 1 conceptual trait associations, B) Study 3 conceptual trait associations averaged across participants. In both A and B only unique values (lower triangles without the diagonal) were used for the analyses. C) Scatterplot between Study 1 and Study 3 conceptual trait associations. Each point represents a trait pair. Line of best fit and 95% confidence intervals plotted. **Face Impressions**: D) Subset of Study 1 face impressions, E) Study 3 face impressions averaged across participants. In both D and E only unique values (lower triangles without the diagonal) were used for the analyses. F) Scatterplot between Study 1 and Study 3 face impressions. Each point represents a trait pair. Line of best fit and 95% confidence intervals plotted.

**Supplementary Materials 4**

Following peer review, we conducted additional exploratory analyses, subjected to FDR correction due to their post-hoc nature. All data and full analysis scripts can be found here: <https://osf.io/bjz8n/?view_only=4a3342013aa0426eb8c82a3f0ca75fb6>.

*4.1. Are Traits ‘Old’ vs ‘Young’ and ‘Masculine’ vs ‘Feminine’ Opposite or Independent Concepts?*

In Study 1 and 2, we collapsed across conceptual associations for traits old and young into ‘age’ and masculine and feminine into ‘masculinity’ by inverting young (feminine) and averaging it with old (masculine). To address whether traits within these pairings are opposites of one another, here we disseminate participant responses to the conceptual association questions “If someone is old (young), how likely are they to be young (old)?” and “If someone is masculine (feminine), how likely are they to be feminine (masculine)?”. These responses ranged from 1 (indicating they are opposites) through 4 (indicating they are unrelated concepts) to 7 (indicating they are very similar).

Perceivers most frequently reported that old/young and masculine/feminine are opposite concepts (most common response was 1) in both Studies 1 & 2 (see **Figure S2**). A Chi-Square test applied to the proportions of participant responses of 1 (concepts are opposite) versus 4 (concepts are unrelated) showed that perceivers significantly more frequently believed old/young are opposite concepts (S1: *X^2^*(1) = 129.75, FDR-corrected *p* < .0001; S2: *X^2^*(1) = 115.04, FDR-corrected *p* < .0001) as well as masculine/feminine are opposite concepts (S1: *X^2^*(1) = 52.982, FDR-corrected *p* < . 0001; S2: *X^2^*(1) = 17.405, FDR-corrected *p* < .0001) rather than unrelated concepts. Thus, this cross-validation suggests that collapsing across conceptual associations of old/young and masculine/feminine is appropriate in both Studies 1 and 2.

**Figure S2**

*British (Study 1) and Chinese (Study 2) Perceivers Consider Old vs Young and Masculine vs Feminine to Be Opposite Concepts*
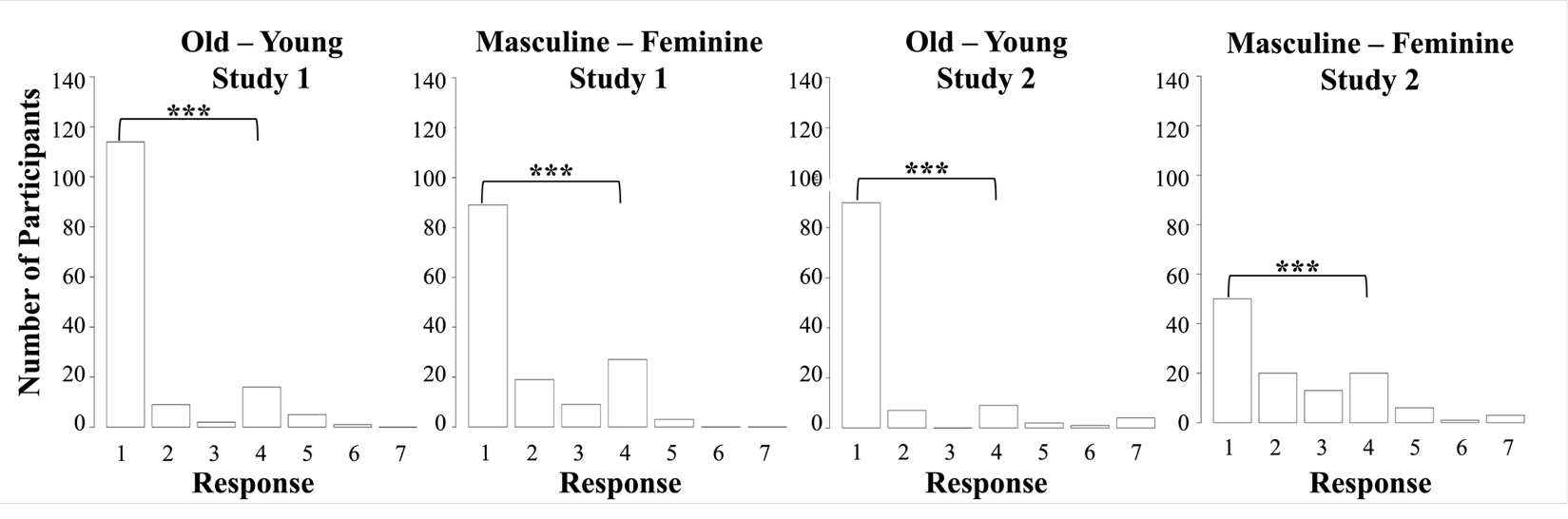


*Note.* Frequencies of each response option (1-7) to conceptual associations questions between old-young (e.g., “If someone is old, how likely are they to be young?”) and masculine-feminine (e.g., “If someone is masculine, how likely are they to be feminine?”). Significance codes *** indicate that the proportion of responses 1 (not at all) to total responses were significantly different from the proportion of responses 4 (neutral) to total responses, as per Chi-squared tests (*p*<.0001 pre and post FDR correction).

*4.2. Inter-Rater Reliability*

We computed Cronbach’s alpha for participants’ conceptual associations and face impressions, separated by perceiver or stimuli sex/gender where applicable.

Conceptual associations had excellent inter-rater reliability across the full participant sample (N_S1_ = 147, N_S2_ = 113; αs > .90), and also individually for female perceivers (n_S1_ = 73, n_S2_ = 64; αs > .90) and male perceivers (n_S1_ = 72, n_S2_ = 47; αs > .90) for both S1 and S2. Note that we did not compute Cronbach’s alphas for S3 as unlike in S1 and S2, perceivers only provided conceptual associations of one trait-pair.

Face impressions across intermixed male and female faces had excellent inter-rater reliability per each trait (αs > .95, S3). Reliability remained excellent for male and female faces separately for each trait (αs >.90, S3). Reliabilities were good for the majority of traits for face and participant sex/gender in face impressions data used in S1 and S2 (as per Sutherland et al., 2018; αs > .70, Tables S1, S2).

*4.3. Perceiver and Face Sex/Gender Differences*

First, we sought to assess whether conceptual associations predict face impressions across participant sex/gender (S1-S2). British female perceivers’ (n = 73) conceptual associations were positively related to British perceivers’ face impressions (RSA, rho (*64*) = .69, uncorrected *p* <.0002, 5,000 permutations; FDR-corrected *p* < .0007), as were British male perceivers’ (n = 72) conceptual associations (RSA, rho(*64*) = .71, uncorrected *p* <.0002, 5,000 permutations; FDR-corrected *p* < .0007). Similarly, Chinese female perceivers’ conceptual associations (n=64) were positively related to Chinese perceivers’ face impressions (RSA, rho(64)=.77, uncorrected p <.0002; FDR-corrected *p* < .0007), as were Chinese male perceivers’ (n = 47) conceptual associations (RSA, rho(64) = .79, uncorrected p<.0002; FDR-corrected *p* < .0007). We found no difference between how female and male perceivers’ conceptual associations predicted face impressions in our British sample (*z* = -.22, uncorrected *p* = .83, FDR-corrected *p* = .86), nor the Chinese sample (*z* = .28, uncorrected *p* = .78, FDR-corrected *p* = .86). Thus, we found no evidence that conceptual associations of male and female perceivers predicted face impressions differently in British and Chinese perceivers. We could not assess the face-level sex/gender differences as we lacked the data.

Second, we compared the relationships between conceptual associations and face impressions in male/female perceivers judging male (n = 50) or female (n = 50) faces on the individual perceiver level (Study 3, see **Figure S3**). Female perceivers’ (n = 114) conceptual associations positively related to their impressions of female faces (rho(*112*) = .26, uncorrected *p* <.006, FDR-corrected *p* < .02). However, there was no significant relationship with impressions of male faces (rho(*112*) = .13, uncorrected *p* =.16, FDR-corrected *p* = .30). Male perceivers’ (n = 119) conceptual associations positively related to their impressions of male faces (rho(*117*) = .50, FDR-corrected *p* < .0001), as well as female faces (rho(*117*) = .45, FDR-corrected *p* < .0001).

Further correlation strength comparisons found no evidence that the relationship between female perceivers’ associations and their impressions of female faces differed from the relationship between male perceivers’ associations and their impressions of male faces following an FDR-correction (z = -2.13, uncorrected *p* < .04, FDR-corrected *p* = .07) nor for female faces (z = -1.64, uncorrected *p* = .099, FDR-corrected *p* = .20). Next, we did not find evidence that male perceivers’ relationship between their conceptual associations and impressions of male versus female faces differed (z = 0.48, uncorrected *p* = .62, FDR-corrected *p* = .74). As the relationship between female perceivers’ conceptual associations and their impressions of male faces was not statistically significant, we did not conduct any additional comparisons. Altogether, these results show that in our S3 sample, there was no evidence that female perceivers’ conceptual associations predicted their impressions of male faces, unlike for other groups. However, there was no evidence that male and female perceivers differed in the extent to which their conceptual associations predicted impressions of male or female faces otherwise.

**Figure S3**

*Relationship Between Conceptual Associations and Face Impressions Split by Perceiver and Face Sex/Gender*


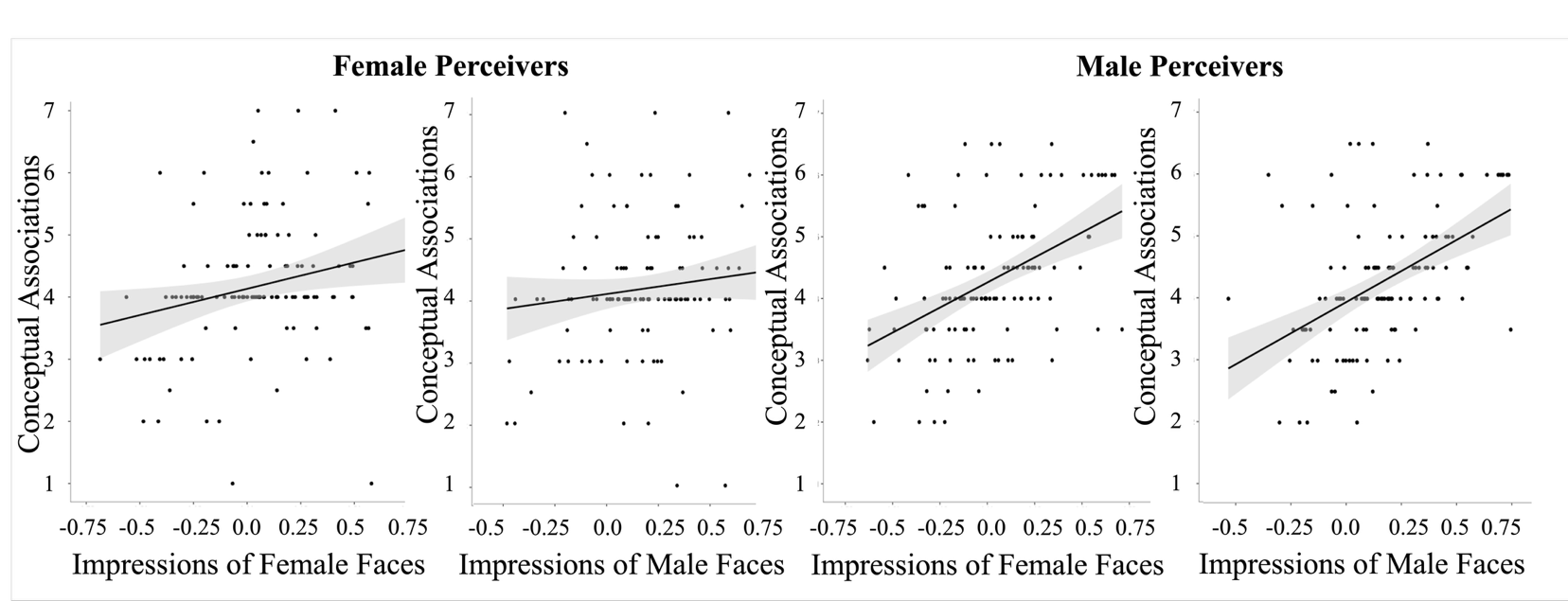


*Note.* Trait pair data points, lines of best fit, and 95% CI are plotted.

*4.4. Trait Pair Differences*

Finally, we address the relationship between conceptual associations and face impressions for each trait pair individually. To do so, we applied Spearman’s correlation to data from Study 3, where each individual perceiver provided conceptual associations and face impressions for one trait pair only, yielding approximately 15 participants (data points) per trait pair group.

First, perceivers’ conceptual associations and face impressions were positively related for intelligent-old (*rho*(*14*) = .733, uncorrected *p* < .002, FDR-corrected *p* = .004), suggesting that perceivers’ conceptual associations between age and intelligence captured their face impressions when rating facial age and intelligence. Second, conceptual associations and face impressions were negatively related for friendly-intelligent (*rho*(*14*) = -0.619, uncorrected *p* < .02, FDR-corrected *p* < .03), and nice-masculine (*rho*(*12*) = -.696, uncorrected *p* < .01, FDR-corrected *p* < .02). These results suggests that those perceivers who, for example, reported that they believed that friendly people are intelligent, rated friendly faces as unintelligent. Finally, Spearman’s correlation did not find evidence for a relationship between conceptual associations and face impressions for other trait pairs (**Table S7**). However, at the sample size of 15 perceivers per trait pair, we had 80% power to detect *rho* = |0.657| at *p* <.05. Given the low statistical power, these supplementary analyses should be interpreted with caution and are presented for information about effect sizes only.

**Table S7**

*Spearman’s Correlation between Conceptual Associations and Face Impressions per Each Trait Pair*

| Trait Pair | Spearman’s *rho* | Raw *p* | FDR-corrected *p* |
| --- | --- | --- | --- |
| attractive-intelligent | 0.151 | 0.589 | 0.726 |
| attractive-masculine | 0.347 | 0.204 | 0.344 |
| attractive-old | 0.185 | 0.476 | 0.624 |
| friendly-attractive | 0.181 | 0.487 | 0.624 |
| friendly-intelligent | -0.619 | 0.011* | 0.024* |
| friendly-masculine | -0.071 | 0.794 | 0.859 |
| friendly-nice | 0.208 | 0.439 | 0.611 |
| friendly-old | -0.058 | 0.832 | 0.344 |
| intelligent-old | 0.733 | 0.001*** | 0.004*** |
| masculine-intelligent | -0.021 | 0.940 | 0.940 |
| masculine-old | 0.265 | 0.321 | 0.467 |
| nice-attractive | 0.305 | 0.289 | 0.454 |
| nice-intelligent | 0.300 | 0.298 | 0.454 |
| nice-masculine | -0.696 | 0.006** | 0.014* |
| nice-old | 0.347 | 0.205 | 0.344 |

*Note.* Significance codes: * <.05 **<.01 ***<.005
